# Hypothetical molecular interconnection between type 2 diabetes and dyslexia

**DOI:** 10.1186/s12868-021-00666-9

**Published:** 2021-10-21

**Authors:** Tuğba Bülbül, Maryam Baharlooie, Zahra Safaeinejad, Ali Osmay Gure, Kamran Ghaedi

**Affiliations:** 1 Department of Biomedical Sciences, Faculty of Health and Life Sciences, Coventry University, Coventry, West Midlands UK; 2 Department of Medical Biology, Acibadem University, Istanbul, Turkey; 3 Department of Molecular Biology and Genetics, Faculty of Science, Bilkent University, Ankara, Turkey; 4 Department of Cell and Molecular Biology and Microbiology, Faculty of Biological Science and Technology, University of Isfahan, Hezar Jerib Avenue, Azadi Sq., P.O.Code 81746-73441, Isfahan, Iran; 5 Department of Animal Biotechnology, Cell Science Research Center, Royan Institute for Biotechnology, ACECR, Isfahan, Iran

**Keywords:** Dyslexia, T2D, Hyperglycaemia, WNT signalling, Primary cilia, *DCDC2*, *ROBO1*, *TCF7L2*, *CTNNB1*

## Abstract

**Background:**

Dyslexia is one of the most common learning disabilities, especially among children. Type 2 diabetes is a metabolic disorder that affects a large population globally, with metabolic disorders. There have been several genes that are identified as causes of Dyslexia, and in recent studies, it has been found out that some of those genes are also involved in several metabolic pathways. For several years, it has been known that type 2 diabetes causes several neurodegenerative disorders, such as Alzheimer’s disease and Parkinson’s disease. Furthermore, in several studies, it was suggested that type 2 diabetes also has some associations with learning disabilities. This raises the question of whether “*Is there a connection between type 2 diabetes and dyslexia*?”. In this study, this question is elaborated by linking their developmental processes via bioinformatics analysis about these two diseases individually and collectively.

**Result:**

The literature review for dyslexia and type two diabetes was completed. As the result of this literature review, the genes that are associated to type 2 diabetes and dyslexia were identified. The biological pathways of dyslexia, and dyslexia associated genes, type 2 diabetes, and type 2 diabetes associated genes were identified. The association of these genes, regarding to their association with pathways were analysed, and using STRING database the gene associations were analysed and identified.

**Conclusion:**

The findings of this research included the interaction analysis via gene association, co-expression and protein–protein interaction. These findings clarified the interconnection between dyslexia and type 2 diabetes in molecular level and it will be the beginning of an answer regarding to the relationship between T2D and dyslexia. Finally, by improving the understanding this paper aims to open the way for the possible future approach to examine this hypothesis.

## Background—Introduction

### Hallmarks of dyslexia, psychology, and behaviour

Dyslexia is a neurobiological disorder that causes a learning disability. It is the most common type of learning disability; however, there are still many unknowns about its neurobiological pathways and how it is related to other risk factors and conditions. Unlike other learning disabilities, Dyslexia does not reflect on the IQ level of the patient [1]. It causes difficulty in reading and writing. Also, if not diagnosed, Dyslexia may lead to low self-esteem in children [1]. It was firstly identified in 1878 by Adolf Kussmaul, who introduced the world with the concept of ‘Word blindness’ through his observations with his stroke patients, who seem to have selectively lost their ability to read but remain their verbal and non-verbal reasoning skills [2]. This condition was later named *Dyslexia* by Rudolph Berlin, and the information on this condition was later extended by Pringle Morgan, who explained ‘congenital word blindness’ through his studies [2]. Until the mid-twentieth century, it was thought that this condition was due to a hereditary defect, however, later, it was found out though it might also be due to hereditary defects, it can also be due to non-hereditary genetic problems. There are several symptoms of Dyslexia, which are observed as linguistic, sensory, and motor signs [3, 4]. The most significant symptom of Dyslexia is difficulty in reading and writing. In developmental Dyslexia cases, the symptoms can be delayed speech or other speech problems such as stuttering, learning and writing difficulty, and visual disturbances while reading, such as blurred vision, double vision, eye strain, headaches and eye fatigue [5].

In 1986, Hansen et al. reported a case study with a group of diabetic patients to find out if there is any association between Dyslexia and Diabetes. They identified the history of Dyslexia in Type 1 Diabetic patients. While some of those patients have been identified as dyslexic, some were not. But they had family members who were dyslexic [6]. In 1987, Hansen, Nerup, and Holbek published a follow up on their research, in which they performed their experiment again to compare the accuracy of their findings. The results were very similar to each other that they concluded their study output, stating that 21% of dyslexic relatives were among 114 diabetics, and in those 114 patients, four were dyslexic. On their findings, there must be a common dominantly inherited autosomal genetic error, which causes both disorders [7]. Upon their research, several other case studies supported their findings by stating there might be some minor or major deficits that may lead to both dyslexia and diabetes [8, 9]. In contrast, those findings were not sufficient enough for them to make a decisive conclusion to state that there is a relationship between Diabetes and Dyslexia.

In the normal human brain, while reading, the left hemisphere of the brain is actively working. The functional difference in the brain of a dyslexia patient, due to the hypoactivity in left temporal, parietal, and fusiform regions sensitivity in reading decreases. It is associated with both anatomical and functional abnormalities in the left hemisphere due to failures in neural migration [10]. In addition to this, there is also a structural difference in the brain of a dyslexia patient, which is the colocalization of the gray matter in lobule VI of the cerebellum, which is involved in the memory and voluntary movements, and the region that has functional differences with normal brain [11]. It affects the daily life of the patient. Nevertheless, there is no functional medicine as a treatment of Dyslexia. However, there are several exercises for the patients with Dyslexia as a treatment, such as reading and writing therapies and spelling therapies.

### Hallmarks of type 2 diabetes, pathophysiology and biology

Diabetes mellitus (DM) is the most common disease among the people in the world [12]. Type 2 diabetes (T2D) Mellitus is one of the most common diabetes types, and nowadays, it has started to spread among young people due to unhealthy lifestyles and inheritance. T2D can be caused by either genetic factors or/and environmental factors. The leading genetic factors behind T2D are impaired insulin secretion or insulin resistance, or it can even be both due to impaired pancreatic β-cells [13, 14]. These lead to a metabolic disorder that can also be caused by dysregulation of carbohydrate, lipid, and protein metabolism in the body [12, 15]. The main symptom of T2D is hyperglycaemia, which is a condition with excessive levels of glucose in the blood. This is followed by frequent urination and thirst, weight change, skin irritation, headache, and vision problems such as blurred vision, dry eye and retinopathy [16, 17]. The leading environmental factors behind T2D can be irresponsible high-calorie diets, excessive glucose consumption, or it can also be due to the lack of physical activity. Because of these conditions, the glucose homeostasis system in the body can be affected, which shows itself as insulin resistance at first; if it is not treated with a healthy diet and physical activity, the condition can develop T2D [18]. However, these factors might also cause a functional problem in pancreatic β-cell secretion or directly on β-cell functioning, leading to T2D [13].

Insulin is secreted by pancreatic β-cells, and due to an impairment in these cells, the insulin secretion is dysregulated. As a result of this condition, the insulin secretion is blocked, and it leads to a hyperglycaemic condition and T1D via pathogenic mechanisms. When the body cannot produce enough insulin to absorb excessive amounts of blood glucose, the body goes into a hyperglycaemic state. When this happens in the patients with T2D, the kidneys and liver play an essential role in compensating or regulating the glucose homeostasis, in which the kidneys and liver try to balance the insulin and glucagon in the blood to maintain blood glucose levels [18]. Another malfunction of T2D is insulin-mediated whole-body glucose utilization, which is due to the defective glucose disposal in the muscle; the amount of glucose intake significantly decreases [8, 18]. These are the two most important problems among T2D patients, controlled by therapeutic diabetes drugs combined with dietary restrictions and increased physical activity. T2D is correlated with aging because as the person ages, the frequency and the intensity of the islet amyloids increase [19]. This increase is mostly associated with the malfunctioning of pancreatic β-cells.

## The hypothesis

According to the initial literature research that was discussed in the introduction, the first similarity between Dyslexia and type 2 diabetes is that they have an important common symptom, vision impairment. Although the reason behind the vision impairment in dyslexia is not clear yet it is hypothesised that the efficiency of ocular motor tracking or other parameters of visual functioning may be the possible cause of it [5]. It has been identified that the visual impairment in T2D is related to microvascular complications, which may cause low blood and fluid flow to the eyes that may lead blurred vision, dry eyes or retinopathy according to the severity of the condition [17]. Due blurred vision that is caused by microvascular complications, reading difficulties can be developed which may trigger Dyslexia, however, it requires further research within the dyslexic patients and their microvascular system. Headache can be a linked cause of blurred vision; however, it could also be the reason of hyperglycaemic condition within the body of T2D patients.

## Molecular mechanisms and related genes

### Physiology and molecular mechanism of dyslexia

The efforts on Dyslexia are currently increasing and evolving. However, we have not yet had sufficient information on Dyslexia. According to the current researches, we are aware that there are some functional and structural differences in the brain of patients with Dyslexia compared to the average population. Norton et al. stated that there are hypoactivation in the left temporal, parietal, and fusiform regions of the brain. Most cases are due to lack of differential sensitivity. Even though it differs among the people according to their age, gender, ethnic diversity, it’s mostly seen in the left hemisphere, especially the left inferior frontal and angular and temporal gyrus [1, 20, 21]. According to Ramus et al., the post-mortem survey, showed that there are structural differences between a person with dyslexic and a control brain, which are seen in the left perisylvian cortex, underlying white matter, thalamus, corpus callosum, and cerebellum. As a result of the researches, the neural signature of Dyslexia is identified as the failure of the left-hemisphere posterior of the brain to function correctly [21].

The findings from several studies indicate that these functional abnormalities are the results of neuronal migration. While in some patients, this abnormal neuronal migration is seen in microgyria as a disturbed organization of all layers of the cortex, in some patients, cytoarchitectonic anomalies, disorganized magnocellular layers with small neuronal cell bodies, in the thalamus is observed [3]. It was observed that the neuronal cell bodies in the magnocellular layers of the lateral geniculate nucleus are smaller in the dyslexic patient with respect to a healthy individual, and the neuronal asymmetry of the primary visual cortex wasn’t seen in dyslexic patients [22]. Another structural difference in the brain of the patients with Dyslexia is the structural white and gray matter difference; in which it has been observed that the gray matter colocalizes with the regions that show functional differences (left hemisphere) as well as the cerebellum, especially in lobule VI [1]. According to the researches that have been done on the white matter of dyslexic patients, the local white matter changes in the left temporoparietal regions and the left interior frontal gyrus [10]. Peterson et al. stated that the gray matter density decreases in the left medial temporal gyrus of dyslexic patients. White matter also decreases in the left frontal and parietal portions of the arcuate fasciculus and other left hemisphere sites in the dyslexic patients. While these decreases result in reading disability, according to researches that have been done until now, there has been no indication of it causing any mental disability.

Over the last few years, several genes that are linked with developmental Dyslexia have been identified. There are six key genes for developing Dyslexia, including *DYX1C1*, *ROBO1*, *DCDC2*, *K1AA0319*, *SLIT1*, and *SRGAP1*. In 1983, Smith et al. represented a new locus, *DYX1* locus, linked with the reading disability, which is later discovered that the gene on that locus (***DYX1C1*** gene) is a genetic risk factor for developmental dyslexia [20, 23]. ***ROBO1*** is a crucial gene for Dyslexia because it encodes an integral membrane protein, which functions in axon guidance as well as neuronal cell migration. It is known as axon guidance receptor gene. During brain development, two functional copies of ***ROBO1*** are required to have an average reading ability; however, in the event when there is a partial haploinsufficiency for ***ROBO1***, the patient develops dyslexia [24]. ***K1AA0319*** gene encodes a transmembrane protein. It regulates the neuronal migration and cell adhesion, which plays a part in the development of the cerebral cortex. ***DCDC2*** gene belongs to a superfamily of doublecortin domain-containing proteins that bind to microtubules, which is involved in neuronal migration, and it is localized to the primary cilium in the primary neurons [25]. Massinen et al. states that while the overexpression of ***DCDC2*** affects the ciliary length and Shh signalling, which is a signalling pathway that functions in the cell differentiation during embryonic development; downregulation of ***DCDC2*** expression enhances WNT signalling, which is a signalling pathway during the embryonic development and the development of the nervous system (neurogenesis). As a result of a disruption in the ***DCDC2*** gene, migration of developing neurons via these pathways from the ventricular zone to their cortical plate is also disrupted and impaired [25]. ***K1AA0319*** is also responsible for the development of Dyslexia, and it functions with ***DCDC2*** because the evidence states that there is a gene–gene interaction between ***K1AA0319*** and ***DCDC2*** [23]. Also, ***SLIT1*** is thought to be associated with the development of dyslexia because it is responsible for preventing unwanted midline crossing of axons by acting as the molecular guidance cue in cellular migration. ***SRGAP1*** is a protein-coding gene which is involved in GTPase activator activity, and it is associated with ***ROBO1*** and ***SLIT*** protein family. However, it is not yet clear how ***SLIT1*** and ***SRGAP1*** are directly involved in the development of dyslexia. ***DYX1C1***, ***ROBO1***, ***DCDC2***, and ***KIAA0319***, are effectively functional in the neuronal migration and axon guidance, and these four genes collectively work while they also co-regulate each other [10]. ***DYX1C1*** and ***DCDC2*** genes are related to ciliary Dyslexia. They are regulated by Regulatory Factor (RF) X transcription factors, whereas ***DYX1C1***, ***DCDC2***, and ***KIAA0319*** are linked to the cytoskeletal organisation, which causes them to work together in the neuronal migration in the brain [25]. As to complement their work, ***ROBO1*** acts as a neuronal axon guidance gene through the neuronal migration [26]. These four genes were the basis of the susceptibility of developing dyslexia genes. In several studies, such as Anthoni et al., they are used as a comparison to analyse other dyslexia-related genes [24].

### Physiology and molecular mechanism of type 2 diabetes

Type 2 diabetes mellitus is the most common type of diabetes disorder. The reasons behind T2D are either defective insulin secretion or loss of glucose uptake in the glucose consuming tissues. As previously mentioned in this article, pancreatic β-cell death is the leading cause of type 2 diabetes. As the functionality of the β-cell decreases, glucose intolerance starts to develop; however, if it progresses drastically, type 2 diabetes is developed [14]. Several studies [14, 27] show that it is also linked with lipotoxicity or glucotoxicity, resulting from over-usage of pancreatic β-cells due to the excessive amount of glucose in the blood. In both cases, hyperglycaemia is the key response element for type 2 diabetes. For *glucotoxicity*, due to the over-usage, the phenotype of β-cells, or glucose stimulus-secretion coupling and even gene expression, can change, which leads to apoptosis of the pancreatic β-cells [27, 28]. In the case of *lipotoxicity*, it has been observed that abdominal obesity is correlated with loss of β-cell function, which leads to glucose intolerance, which also results in inflammatory response [14, 27]. This apoptosis can also be a result of inflammatory reactions when there are high concentrations of *proinflammatory* cytokinesis because of the excessive levels of glucose in the blood [28].

*Hyperglycaemia* is the leading condition of type 2 diabetes, and as it was previously mentioned that hyperglycaemia is affected by the volume of fat tissue in the body. Glucose homeostasis is the balance between the insulin and the glucagon in the blood to preserve the blood glucose levels, and it is associated with peripheral insulin resistance. For glucose homeostasis, the adipose tissue is vital because by producing cytokines and several other inflammatory pathway reagents, the white adipose tissue is the primary source of inflammatory markers in T2D [29]. The free fatty acids lead to chronic metabolic inflammation, which leads to endoplasmic reticulum (ER) stress, TLR4 signalling, protein kinase Cε or protein kinase R(PKR), and insulin resistance [30]. In order to preserve the homeostasis, inflammation responses are triggered, which results in apoptosis [30].

Aggregating researches provided us with the genetic information of type 2 diabetes. There are several risk-factor genes involved in the development and the progress of type 2 diabetes. T2D can be caused by a mutation in genes involved in metabolic pathways, such as a single polymorphism (SNP) in *TCF7L2* gene [12]. T2D also be caused by chronic inflammation due to proteins in inflammatory pathways, such as interleukin-6 (IL-6) and tumour necrosis factor (TNF) [12]. *TCF7L2* is the most known of those genes, which are associated with type 2 diabetes. It encodes a high mobility group (HMG) box-containing transcription factor, which is involved in regulating blood glucose homeostasis, and it is associated with the increased risk of developing T2D. It is also essential for the *WNT signalling* pathway, which is a key pathway that functions in organogenesis and the development and the process of tumours during embryonic development, and it is also involved in regulating gene expression during the adulthood [23]. Transcription factor β-catenin (β-catenin)/TCF acts as an effector for the canonical signalling pathway; in the absence of WNT signalling, HMG box TCF proteins functions as the transcriptional corepressors of the WNT target genes in the nucleus [23, 31]. β-catenin governs the development of pancreatic islet and with the adipocyte-derived WNT molecules induces the pancreatic β-cell proliferation, and insulin secretion [31]. It has been observed and reported in several studies that due to polymorphism in *TCF7L2*, the risk of developing type 2 diabetes significantly increases. According to the researches, SNPs on *TCF2L7* cause insulin secretion level reduction [23]. However, according to the study of Shu et al., when *TCF7L2* is overexpressed, the islets are prevented from glucose and cytokine-mediated apoptosis of pancreatic β-cells.

Another key gene for type 2 diabetes is *CTNNB1*. It is responsible for encoding β-catenin, and it is involved in cadherin-mediated intracellular adhesion and cell growth. It is actively involved in the WNT signalling pathway. Upon the activation of WNT/CTNNB1 signalling, Pyruvate kinase muscle isoform 2 (PKM2), a potential modulator of insulin secretion in pancreatic β-cells, inhibits cell apoptosis and simultaneously promotes cell proliferation, as well as insulin secretion [31]. In addition to this, ***KIF3A,*** which is a kinesin motor protein and an essential subunit for the transportation along microtubules in cilium and cytoplasm in the neurons, is also a critical gene in the development of type 2 diabetes [25]. The critical component of *KIF3* is necessary for *GLUT4* translocation, and by forming a complex with Axin and TNKS, *KIF3A* is directly involved in glucose transportation by promoting *GLUT4* translocation [32].

## Evaluation of the hypothesis

### Interconnection between dyslexia and T2D molecular mechanisms

According to Massinen et al., overexpression of *DCDC2* may enhance *KIF3A* mediated translocation of the transmembrane protein Smoothened (Smo) to the cilium and leads to overactivation of the Shh signalling pathway. As it was mentioned before, KIF3A is involved in the regulation of phosphorylation and stabilization of β-catenin, which interacts with TCF family proteins, which are associated with the increased risk of developing type 2 diabetes [32]. With this finding, the knowledge about the association between type 2 diabetes and Dyslexia deepened. However, the literature mentioned above, didn’t provide detailed information because there is insufficient data about this association [25].

As previously mentioned, with the literature research on both the common and rare genetic backgrounds of Dyslexia and type 2 diabetes, it was identified that both disorders are genetically linked with the canonical WNT signalling pathway. WNT signalling pathway is an evolutionarily conserved pathway, which regulates cell migration, cell fate determination, cell polarity, neural patterning, and organogenesis during embryonic development, and it is divided into two types as canonical and non-canonical pathways [33, 34]. The canonical pathway is essential for cell fate determination during the early embryogenesis, and the accumulation and translocation of the adherents junction-associated β-catenin into the nucleus is the hallmark of this pathway [33]. Regulation of β-catenin is essential for both dyslexia and type 2 diabetes. While the overexpression of DCDC2 inhibits β-catenin-dependent WNT signalling, the upregulation of β-catenin levels may lead to an increase in the INS-1 cell. As it was mentioned before, DCDC2 is an essential gene for developmental Dyslexia, and it is localized to the ciliary axoneme and the mitotic spindle fibres in a cell-cycle-dependent manner [35]. When DCDC2 is knocked down, the number of cilia in the cell culture reduces [35], leading to developmental Dyslexia. It was also discovered previously that β-catenin/TCF7L2-dependent WNT signalling is involved in the islet function, the development of the pancreas, and the production and the secretion of insulin [36, 37]. When TCF7L2 increases in the islets, insulin secretion can be inhibited, which leads to an increased risk of diabetes [36]. In addition to this modulation of β-cell growth is affected by E-cadherin, which forms a link with actin by β-catenin. Due to the problem in β-catenin, the risk of developing type 2 diabetes can increase as well [37, 38].

### Interaction analysis—results

**Fig. 1:**
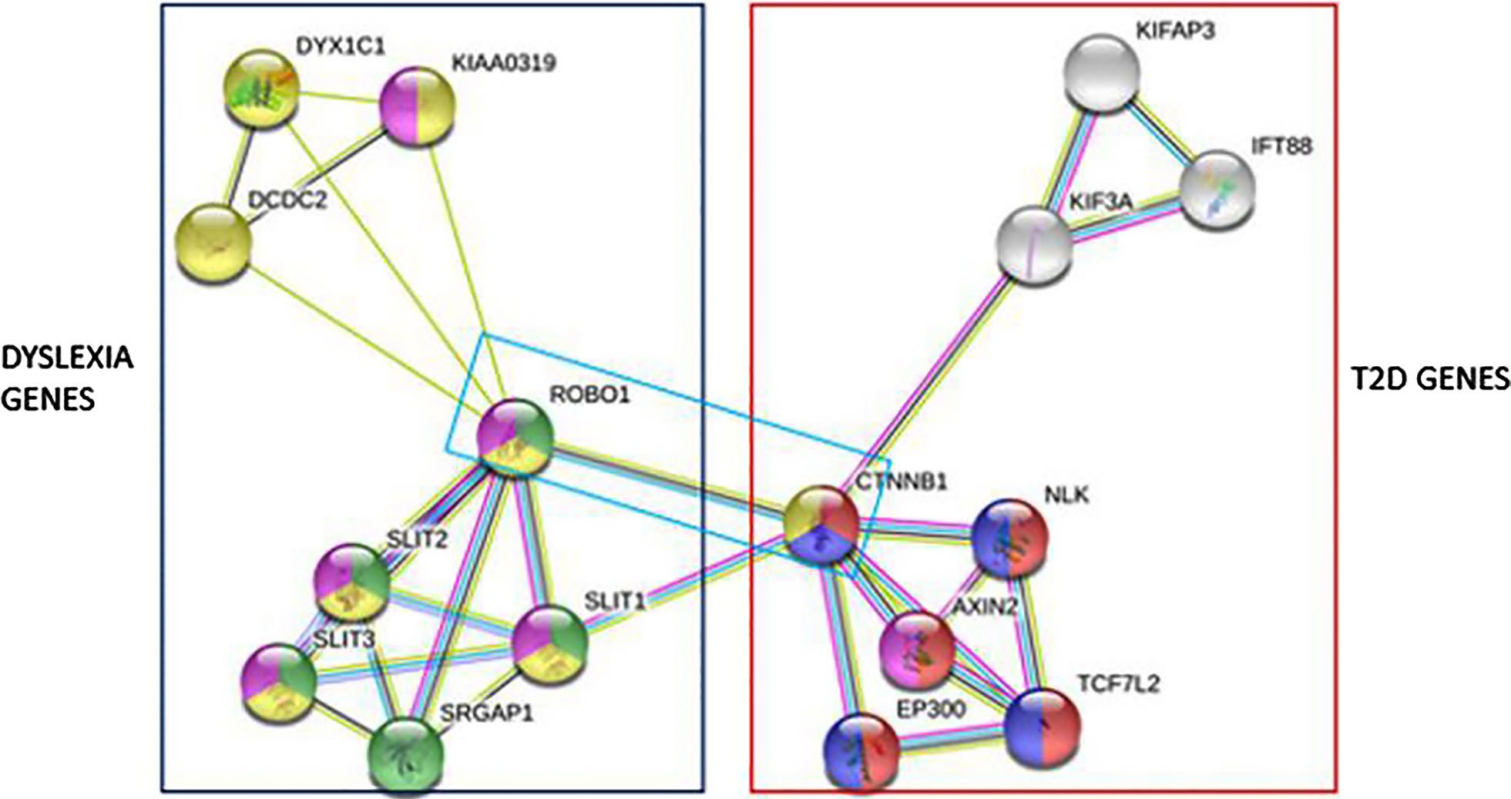
Protein interaction network obtained from STRING database. In the left side of the figure the genes that are related to dyslexia are given, and on the right side of the figure the genes that are associated with type 2 diabetes are shown. The light-blue lines between the genes indicates interactions from curated database, while the pink lines between the genes indicates interactions that are experimentally determined. Green lines between the genes indicates predicted interactions of gene neighbourhood, while red lines indicate gene fusions and dark-blue lines indicates gene co-occurrence. Finally, the yellow lines indicate textmining co-occurrence, black lines indicate co-expression and blue-greyish lines indicates protein homology. These indications are also explained at the legend of the figure. As it is shown in a light blue box, the important connection between dyslexia and type 2 diabetes is between ROBO1 and CTNNB1, from curated database, textmining and co-expression

Upon the findings from the literature, the genes involved in the development of Dyslexia and diabetes, separately, were gathered and divided into two gene sets as Dyslexia genes and T2D genes. In order to understand their protein–protein interactions, the genes that are shown in Fig. 1 were the inputs and the predicted associations. According to Fig. 1, *ROBO1*, Dyslexia related gene, and *CTNNB1*, type 2 diabetes-related gene, are associated via co-expression; with the known interactions from the curated database. In addition to that, it is also seen in Fig. 1 there is another association between *SLIT1*, which acts as a molecular guidance cue in cellular migration, and *CTNNB1* via interactions from both curated database and experimentally determined; as well as *SLIT1* and *ROBO1* via co-expression with known interactions from both a curated database and experimentally determined. All of the mentioned interactions were identified in textmining too. Figure 1 is a proof that there is protein–protein interaction between these two gene sets via several genes.

**Fig. 2:**
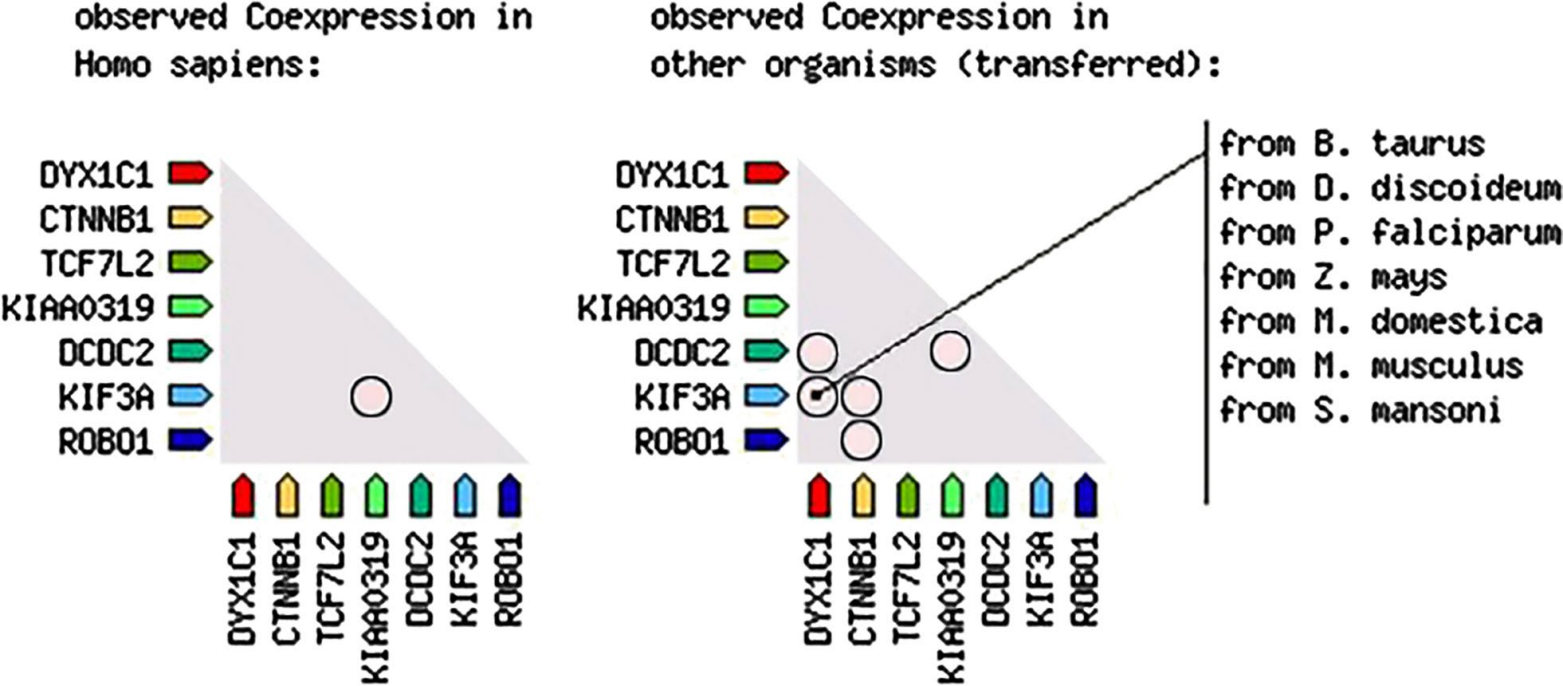
Dyslexia and T2D gene co-expression analysis data from STRING Database. The co-expression of genes that are related to dyslexia and the genes that are related to type 2 diabetes results from STRING Database are shown in this figure. The co-expression of the genes is represented as a heat map. In this figures, light-pink boxes, indicate weak co-expressions between the genes KIAA0319 and KIF3A in Homo sapiens, in the left side of the figure. On the right side of the figure, the co-expression between in other organisms are shown. *S. mansoni* shows a co-expression of ROBO1 and CNTNB1; *M. musculus* shows co-expression of KIF3A, DYX1C1 and CTNNB1; *M. domestica* shows co-expression of DCDC2, DYX1C1 and KIAA0319. The heat map illustrates Co-expression scores based on RNA expression patterns and protein co-regulation, provided by ProteomeHD database and visualized by string database

In addition to the protein–protein interaction network, co-expression of these genes is also queried via the STRING database, which results is represented in Fig. 2. Since these co-expression levels are defined in the STRING database as a heat map, the red squares indicate the co-expression. As it is seen here, there is no strong co-expression evidence between these genes. However, on the left side of the figure, in *Homo sapiens*, a light-pink box is seen between *KIAA0319* and *KIF3A*. This indicates a weak co-expression level. In addition to this, on the right side of the figure, the observed co-expressions in other organisms are shown. As in *S. mansoni*, between *ROBO1* and *CNTNB1*, a weak co-expression is shown, while in *M. musculus*, between *KIF3A* and *DYX1C1* and *KIF3A* and *CTNNB1,* there are light-pink boxes, which indicates a weak co-expression between those genes. Finally, in *M. domestica*, between *DCDC2* and *DYX1C1*, and between *DCDC2* and *KIAA0319*, weak co-expression is shown as light-pink boxes. This figure can also be supportive evidence for the idea of this study and it provides information for possible further research with animal models.

### Flowchart of the research

First, a careful literature search for both diseases was done. More than 30 research papers were screened for each condition individually. During the literature research, the focus was on type 2 diabetes, so the other types of diabetes are not included in this examination. Out of the genes that were mentioned, analysed, and searched in those papers, which are the references, the genes that were related to the developmental stages of each disorder were separated. They are explored in detail, the pathways they are involved in, their functions and their molecular background were searched. With these data, the genes that are associated with Dyslexia and genes that are associated with type 2 diabetes are sorted and listed. These genes are entered into the STRING database for protein–protein interactions [39]. After the initial input, predicted functional partners were shown. The network settings are chosen as network edges to represent the type of evidence. The active interaction sources are chosen as text mining, experiments, database, co-expression, neighbourhood, gene fusion, and co-occurrence. The minimum required interaction score was chosen as 0.400 for medium confidence. Finally, the max number of interactions to show are selected as no more than ten interactions.

## Consequences of the hypothesis, discussion and further aspects

In the past, it was thought that Dyslexia is caused by an underlying deficit in phonological representations. Still, now it is known that a single phonological deficit is not enough to diagnose a patient as dyslexic [10]. There are several clinical features of Dyslexia, such as attention and hyperactivity, motor integration disorder, arithmetic, mild oral language, and the severity of these deficits’ changes in patients [40]. On the other hand, Dyslexia is often confused with attention deficit hyperactivity disorder (ADHD) [10]. There are significant differences between these two diseases. For instance, ADHD patients have short focus periods, whereas dyslexia patients can maintain their focus; however, they have difficulty in reading, writing a word, or a number correctly.

In this research, the aim was to analyse if there is an association between type 2 diabetes and Dyslexia at the molecular level since type 2 diabetes is associated with several neurodegenerative disorders and learning disabilities. According to the literature study, some genes that are associated with developmental dyslexia, such as *ROBO1*, *DCDC2*, *DYX1C1,* and *KIA0319*, and some genes that are associated with type 2 diabetes *CTNNB1*, *TCF7L2,* and *KIF3A* had shown some connections with each other. When these connections are searched further with STRING analyses, the outcome was promising, even though it was small evidence. In Fig. 1, the interactions between *KIAA0319* and *ROBO1* are text mining and co-expression, but they are not experimentally validated, which means that this interaction is less likely to be considered. However, the interaction between *SLIT1* and *CTNNB1* is not only from the curated database, but it is experimentally validated. So, this might be useful evidence for continuing of this research. As it was mentioned above, in the previous studies by Hansen and other researchers, a connection between type 1 diabetes and Dyslexia, was also mentioned. For the future aspect of this research, the association might be improved into the association between diabetes mellitus and Dyslexia upon gathering enough evidence. This research will continue with in vivo analysis.

## Data Availability

All data generated or analysed during this study are included in this published article.

## Abbreviations

ADHD: Attention deficit hyperactivity disorder
CTNNB1: Catenin (cadherin-associated protein) beta 1
DCDC2: Doublecortin domain-containing protein 2
DM: Diabetes mellitus
DYX1: Dyslexia susceptibility 1
DYX1C1: Dyslexia susceptibility 1 candidate gene 1 protein
ER: Endoplasmic reticulum
GLUT4: Human glucose transporter 4
GTPase: Guanosine triphosphate enzyme
HMG: High mobility group
IL-6: Interleukin-6
KIAA0319: Dyslexia-associated protein
KIF3: Kinesin superfamily protein 3
KIF3A: Kinesin family member 3A
PKM2: Pyruvate kinase muscle isoform 2
PKR: Protein kinase R
RF: Regulatory factor
ROBO1: Roundabout guidance receptor 1
SLIT1: Slit guidance ligand 1
Smo: Smoothened (transmembrane protein)
SNP: Single polymorphism
SRGAP: SLIT-ROBO Rho GTPase-activating protein
STRING: Search tool for the retrieval of interacting genes/proteins
T1D: Type 1 diabetes
T2D: Type 2 diabetes
TCF β-catenin: Transcription factor β-catenin
TCF7L2: Transcription factor 7 like 2
TLR4: Toll-like receptor 4
TNF: Tumour necrosis factor
TNKS: Tankyrase
WNT Signalling: Wingless/integrated signalling
